# Supplementary material for: Research on Niche Evaluation of Photovoltaic Agriculture in China
Source: Int J Environ Res Public Health. 2022 Nov 9;19(22):14702. doi: 10.3390/ijerph192214702 (PMC9690549; doi:10.3390/ijerph192214702)
Supplement: Supplementary file 1 [file ijerph-19-14702-s001.zip › ijerph-2016495-supplementary.pdf]

Questionnaires

Dear experts,

I’m sorry to bother you.

I am currently studying the issue of the ecological niche of photovoltaic agriculture. I hope to conduct in-depth research through the analysis of the development status of this innovative model, and put forward corresponding countermeasures and suggestions for the sustainable development of China's photovoltaic agriculture.

This survey is an academic research. All the survey contents and results will be strictly confidential and will not be used for other purposes. Please rest assured and answer as objectively as possible. Thank you for your support!

Questionnaire 1. Scoring Table for Decision-making Information of Niche Evaluation of Photovoltaic Agriculture.

The following 32 elements are the indicators selected by the research team that can preliminarily evaluate the photovoltaic agricultural niche. Please score the importance of these indicators. The score range is [0,5]. Take an integer. The larger the score, the better the indicator reflects the development of photovoltaic agriculture.

Primary indicators	Secondary indicators	Score	Primary indicators	Secondary indicators	Score
Resource niche	Agricultural natural resources		Environmental niche	Conservation of fossil resources	
	Market resources			Microclimate environment improvement	
	Capital resources			Air quality improvement	
	Light resources			Greenhouse gas emission reduction	
	Human resources			Conservation of water and soil	
Technology niche	Scientific research institutions		Social niche	Driving the development of smart agriculture	
	Academic conferences			Structure optimization of energy industry and agriculture	
	Academic papers			Alleviating the contradiction between supply and demand for clean energy	
	Invention patents			Promoting food security	
	Technical improvement			Ensuring energy security	
	Technical norms			Providing employment	
Policy niche	Agriculture policy		Economic niche	Increase in land output	
	Special policy			Agricultural output value	
	Financial policy			Promoting the development of related industries	
	Land use policy			Output value of tourism	
	Photovoltaic industry policy			Output value of photovoltaic power generation	

Questionnaire 2. Scoring Table for Influencing Factors of Indicators.

Please score the interaction degree of the following elements (row elements to column elements). 0 is no effect, 1 is very weak, 2 is weak, 3 is strong, 4 is very strong.

Scoring Table for Interaction of Each Element of The Primary Indicators.

Primary indicators	Resources	Technology	Policy	Environment	Society	Economy
Resources						
Technology						
Policy						
Environment						
Society						
Economy						

Scoring Table for Interaction of Each Element of The Secondary Indicators.

Secondary indicators	Agricultural natural resources	Market resources	Capital resources	Human resources	Scientific research institutions	Academic papers	Invention patents	Technical norms	Agriculture policy	Special policy	Land use policy	Photovoltaic industry policy	Conservation of fossil resources	Microclimate environment improvement	Greenhouse gas emission reduction	Conservation of water and soil	Driving the development of smart agriculture	Structure optimization of energy industry and agriculture	Alleviating the contradiction between supply and demand for clean energy	Ensuring energy security	Increase in land output	Agricultural output value	Promoting the development of related industries	Output value of photovoltaic power generation
Agricultural natural resources																								
Market resources																								
Capital resources																								
Human resources																								
Scientific research institutions																								
Academic papers																								
Invention patents																								
Technical norms																								
Agriculture policy																								
Special policy																								
Land use policy																								
Photovoltaic industry policy																								
Conservation of fossil resources																								
Microclimate environment improvement																								
Greenhouse gas emission reduction																								
Conservation of water and soil																								
Driving the development of smart agriculture																								
Structure optimization of energy industry and agriculture																								
Alleviating the contradiction between supply and demand for clean energy																								
Ensuring energy security																								
Increase in land output																								
Agricultural output value																								
Promoting the development of related industries																								
Output value of photovoltaic power generation																								

Questionnaire 3. Niche Evaluation Rating Form.

The niche comment set of China's photovoltaic agriculture is V = very low, low, medium, high, very high. Now we want to digitize these comment sets. Please assign a value to the grade of the niche level. The interval is [0,5]. Decimals can be taken. The smaller the score is, the lower the niche level is. The larger the score is, the higher the niche level is.

Grades of the niche level	Very low	Low	Medium	High	Very high
Score					

Questionnaire 4. Niche Scoring Table of Indicators.

Please score the ecological niche of indicators according to the assignment criteria of the comment set. The interval is [0,5], and the decimal value can be taken.

Primary indicators	Secondary indicators	Score	Primary indicators	Secondary indicators	Score
Resource niche	Agricultural natural resources		Environmental niche	Conservation of fossil resources	
	Market resources			Microclimate environment improvement	
	Capital resources			Greenhouse gas emission reduction	
	Human resources			Conservation of water and soil	
Technology niche	Scientific research institutions		Social niche	Driving the development of smart agriculture	
	Academic papers			Structure optimization of energy industry and agriculture	
	Invention patents			Alleviating the contradiction between supply and demand for clean energy	
	Technical norms			Ensuring energy security	
Policy niche	Agriculture policy		Economic niche	Increase in land output	
	Special policy			Agricultural output value	
	Land use policy			Promoting the development of related industries	
	Photovoltaic industry policy			Output value of photovoltaic power generation	
